# Early Porcine Sapovirus Infection Disrupts Tight Junctions and Uses Occludin as a Coreceptor

**DOI:** 10.1128/JVI.01773-18

**Published:** 2019-02-05

**Authors:** Mia Madel Alfajaro, Eun-Hyo Cho, Deok-Song Kim, Ji-Yun Kim, Jun-Gyu Park, Mahmoud Soliman, Yeong-Bin Baek, Chul-Ho Park, Mun-Il Kang, Sang-Ik Park, Kyoung-Oh Cho

**Affiliations:** a Laboratory of Veterinary Pathology, College of Veterinary Medicine, Chonnam National University, Gwangju, Republic of Korea; b Chonnam National University Veterinary Teaching Hospital, Gwangju, Republic of Korea; Instituto de Biotecnologia/UNAM

**Keywords:** cellular coreceptor, occludin, sapovirus, tight junction

## Abstract

The genus *Sapovirus* belongs to the family *Caliciviridae*, and its members are common causative agents of severe acute gastroenteritis in both humans and animals. Some caliciviruses are known to use either terminal sialic acids or histo-blood group antigens as attachment factors and/or cell surface proteins, such as CD300lf, CD300ld, and junctional adhesion molecule 1 of tight junctions (TJs), as receptors. However, the roles of TJs and their proteins in sapovirus entry have not been examined. In this study, we found that porcine sapovirus (PSaV) significantly decreased transepithelial electrical resistance and increased paracellular permeability early in infection of LLC-PK cells, suggesting that PSaV dissociates TJs of cells. This led to the interaction between PSaV particles and occludin, which traveled in a complex into late endosomes via Rab5- and Rab7-dependent trafficking. Inhibition of occludin using small interfering RNA (siRNA), a specific antibody, or a dominant-negative mutant significantly blocked the entry of PSaV. Transient expression of occludin in nonpermissive Chinese hamster ovary (CHO) cells conferred susceptibility to PSaV, but only for a limited time. Although claudin-1, another TJ protein, neither directly interacted nor was internalized with PSaV particles, it facilitated PSaV entry and replication in the LLC-PK cells. We conclude that PSaV particles enter LLC-PK cells by binding to occludin as a coreceptor in PSaV-dissociated TJs. PSaV and occludin then form a complex that moves to late endosomes via Rab5- and Rab7-dependent trafficking. In addition, claudin-1 in the TJs opened by PSaV infection facilitates PSaV entry and infection as an entry factor.

**IMPORTANCE** Sapoviruses (SaVs) cause severe acute gastroenteritis in humans and animals. Although they replicate in intestinal epithelial cells, which are tightly sealed by apical-junctional complexes, such as tight junctions (TJs), the mechanisms by which SaVs hijack TJs and their proteins for successful entry and infection remain largely unknown. Here, we demonstrate that porcine SaVs (PSaVs) induce early dissociation of TJs, allowing them to bind to the TJ protein occludin as a functional coreceptor. PSaVs then travel in a complex with occludin into late endosomes through Rab5- and Rab7-dependent trafficking. Claudin-1, another TJ protein, does not directly interact with PSaV but facilitates the entry of PSaV into cells as an entry factor. This work contributes to our understanding of the entry of SaV and other caliciviruses into cells and may aid in the development of efficient and affordable drugs to treat SaV infections.

## Abstract (precis)

Sapoviruses (SaVs) cause severe acute gastroenteritis in humans and animals. Although they replicate in intestinal epithelial cells, which are tightly sealed by apical-junctional complexes, such as tight junctions (TJs), the mechanisms by which SaVs hijack TJs and their proteins for successful entry and infection remain largely unknown. Here, we demonstrate that porcine SaVs (PSaVs) induce early dissociation of TJs, allowing them to bind to the TJ protein occludin as a functional coreceptor. PSaVs then travel in a complex with occludin into late endosomes through Rab5- and Rab7-dependent trafficking. Claudin-1, another TJ protein, does not directly interact with PSaV but facilitates the entry of PSaV into cells as an entry factor. This work contributes to our understanding of the entry of SaV and other caliciviruses into cells and may aid in the development of efficient and affordable drugs to treat SaV infections.

## INTRODUCTION

An essential function of the gut mucosal epithelium is to act as a barrier between the luminal contents and underlying tissue that allows the transport of nutrients, water, and electrolytes but restricts the passage of potentially harmful luminal products and pathogens (1). This barrier is maintained by cell-cell contacts formed by apical-junctional complexes comprising tight junctions (TJs), adherent junctions, and desmosomes (1). TJs, found in epithelial and endothelial cells, are highly specialized intercellular adhesion complexes that contribute to the paracellular barrier (2–4). Numerous studies have unearthed multiple integral membrane proteins located at TJs, including occludin, claudins, junctional adhesion molecules (JAMs), tricellulin, and coxsackievirus and adenovirus receptor (CAR) proteins, as well as their membrane-associated scaffold proteins, such as zona occludens protein 1 (ZO-1), ZO-2, and ZO-3 (2–4). Disruption of TJs results in a decrease in transepithelial electrical resistance (TER) and an increase in paracellular permeability (5) and is associated with various diseases, including cancers, inflammatory bowel disease, and vasogenic edema (2, 6, 7). Although TJs prevent pathogens from crossing the epithelial barrier, they are often targeted by pathogenic viruses and bacteria, leading to infection and the development of disease (7–9). Numerous viruses have developed different mechanisms to subvert host TJ barrier function for their own benefit, such as direct reorganization or degradation of specific TJ proteins, reorganization of the cell cytoskeleton, and activation of host cell signaling events (4, 7). Moreover, some viruses have been found to hijack TJ proteins as receptors for entry and infection (4, 7, 8). For example, rotavirus disrupts TJs and uses the TJ protein JAM-A as a coreceptor (5, 10). The amino-terminal domain of JAM-A also serves as a receptor for prototype and field isolate strains of mammalian reovirus (11, 12). Hepatitis C virus (HCV) interacts with the N-terminal third of extracellular loop 1 in claudin-1, using it as a coreceptor after binding and interaction with the coreceptor CD81 (13, 14). Coxsackievirus and adenovirus use the coxsackievirus and adenovirus receptor protein for their internalization (15, 16). In addition, some viruses do not recognize TJ proteins as entry receptors but instead use them as entry factors to facilitate their entry and infection (10, 17, 18).

Viruses in the family *Caliciviridae* are small, nonenveloped, and icosahedral, and they carry single-stranded, positive-sense genomic RNA (19). The family has five well-established genera: *Norovirus*, *Sapovirus*, *Nebovirus*, *Lagovirus*, and *Vesivirus* (19, 20). Recently, six new genera, tentatively named *Recovirus* (21), *Valovirus* (22), *Bavovirus* (23, 24), *Nacovirus* (24–26), *Salovirus* (27), and *Sanovirus* (28), have been added as unclassified caliciviruses. The most notable genera in the family are *Norovirus* and *Sapovirus*, which cause severe acute gastroenteritis in humans and animals, with noroviruses alone causing ∼200,000 deaths per annum in children <5 years of age (29, 30). Despite their socioeconomic impact, our understanding of the life cycles of these viruses and the development of effective interventions against them have been hampered by a lack of efficient cell culture systems, particularly for those caliciviruses that infect humans (31–33). The Cowden strain of porcine sapovirus (PSaV) is the only strain in the genus *Sapovirus* that replicates in a porcine kidney cell line in the presence of porcine intestinal contents or bile acids, specifically, glycochenodeoxycholic acid (GCDCA) (33, 34). Therefore, PSaV strain Cowden is an attractive model for the study of *Sapovirus* and *Norovirus* life cycles and for use in developing vaccine or therapeutic interventions against human and animal sapoviruses.

Viruses generally initiate infection by binding of virus particles to a specific receptor(s) on the host cell surface (35). Some members of the family *Caliciviridae* have been reported to use either cell surface terminal sialic acids (SAs) or histo-blood group antigens (HBGAs) as attachment factors to facilitate cell binding (36, 37). Recent reports have indicated that, in addition to the use of glycans as attachment factors (38, 39), murine noroviruses (MNoVs) utilize proteinaceous cellular receptors, CD300lf and/or CD300ld (40, 41). Moreover, feline calicivirus (FCV), in the genus *Vesivirus*, which uses α2,6-linked SA as an attachment factor, is also known to use JAM-1, a TJ molecule, as an entry receptor (42, 43). Hom-1 vesivirus, which was isolated from a human patient accidentally infected with the animal calicivirus San Miguel sea lion virus, also recognizes JAM-1 as an entry receptor (44).

Previously, we reported that both α2,3- and α2,6-linked SAs on O-linked glycoproteins act as attachment factors for PSaV entry and infection (37). However, little is known about the roles of TJ proteins in PSaV entry and infection. This prompted us to investigate the potential roles of TJs and their molecules in PSaV entry and infection. In the present study, we found that PSaV strain Cowden dissociates TJs, as indicated by decreased TER and increased paracellular permeability in the porcine kidney cell line LLC-PK. PSaV was observed to directly bind to occludin and then to become internalized as a complex into cells. In addition, these PSaV-occludin complexes were found to travel from early to late endosomes, mediated by small GTPases, Rab5 and Rab7. Our findings indicate that PSaV exploits occludin in epithelial cells to enter and initiate virus replication.

## RESULTS

### PSaV-induced early dissociation of TJs disrupts their functions.

**FIG 1:**
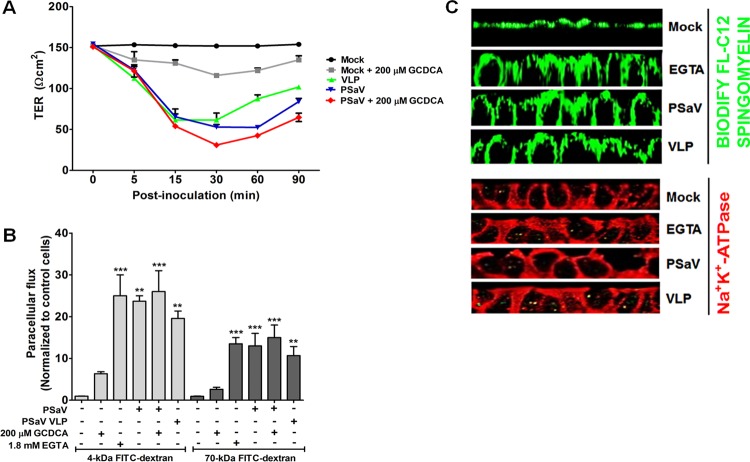
Early PSaV infection dissociates tight junctions. (A) TER was determined in confluent LLC-PK monolayers grown on transwell inserts treated with medium only (mock-treated group) or with 200 μM GCDCA bile acid and infected or not with PSaV strain Cowden (MOI = 50) or PSaV VLPs (4 μg/ml) at the indicated time points. The data are presented as means and standard errors of the mean. (B) Paracellular flux of 4-kDa FITC-dextran and 70-kDa FITC-dextran was measured in the apical-to-basolateral direction of the confluent LLC-PK cells grown on transwell inserts treated or not with 200 μM GCDCA bile acid and the calcium ion chelating agent EGTA (1.8 mM) and infected or not with PSaV strain Cowden (MOI = 50) in the presence or absence of 200 μM GCDCA or PSaV VLP (4 μg/ml). The amount of tracer diffusion was normalized to that of mock-treated, mock-inoculated control LLC-PK cells. The error bars indicate standard errors of the mean. (C) LLC-PK cells grown on the transwell inserts were mock treated (medium only) as a negative control, treated with 1.8 mM EGTA and incubated for 10 min, or infected with the PSaV strain Cowden (MOI = 50) in the presence of 200 μM GCDCA or PSaV VLPs (4 μg/ml) and incubated for 1 h. The distribution of the membrane fluorescent marker Bodify FL-C12-sphingomyelin–BSA (5 nmol/ml) loaded onto the apical surfaces of the LLC-PK cells and detection of the basolateral marker Na^+^K^+^-ATPase were determined by z-sectioning on a confocal microscope. One representative set of results is shown. All experiments were performed in triplicate. **, *P* < 0.001; ***, *P* < 0.0001.

There is increasing evidence that some enteric viruses, such as rotavirus and porcine epidemic diarrhea coronavirus (PEDV), can hijack TJs and their proteins for efficient entry and infection (4, 7, 8, 10, 17, 45, 46). Since TJs are located at the basolateral surfaces of cells and their proteins seal cell-cell adhesion (47), these viruses must dissociate TJs prior to their entry through the basolateral surface of a cell. This in turn results in decreased TER and increased paracellular permeability (5). Therefore, we first examined whether PSaV infection induces a decrease in TER in LLC-PK cells grown on transwell filters. As shown in Fig. 1A, a rapid decrease in TER was observed at 5 min postinfection (p.i.) with PSaV in LLC-PK monolayers without GCDCA supplementation. Supplementation with GCDCA further decreased TER in both virus-inoculated and mock-inoculated cells (Fig. 1A). We further examined whether purified PSaV virus-like particles (VLPs) alone can also open tight junctions. Addition of 4 μg/ml of PSaV VLPs significantly reduced the TER in a manner similar to that of the purified PSaV particles (Fig. 1A). These results suggest that PSaV can dissociate the lateral surfaces of LLC-PK cells early in infection and that addition of bile acids enhances the disruption of TJs.

One of the functions of TJs is to serve as a selective barrier for entry of nonionic molecules via the paracellular route (5, 48). Because PSaV induced dissociation of TJs very early in infection, we next examined whether PSaV infection affected the passage of two nonionic tracers (4-kDa and 70-kDa fluorescein isothiocyanate [FITC]-dextrans) in cells grown on transwell filters (Fig. 1B). LLC-PK monolayers treated without any chemical (the mock-treated group) showed minimal diffusion of these tracers across the monolayers, confirming intact TJs (Fig. 1B). As a positive control, treatment of LLC-PK cells with the calcium ion chelating agent EGTA, known to open TJs and generate epithelial depolarization (5, 49, 50), induced a 25-fold increase in 4-kDa FITC-dextran paracellular diffusion and a 15-fold increase in 70-kDa FITC-dextran paracellular diffusion compared to mock-treated cells (Fig. 1B). Incubation of PSaV in cell culture for 30 min without GCDCA supplementation produced a 22-fold increase in the diffusion of 4-kDa FITC-dextran and a 12-fold increase in the diffusion of 70-kDa FITC-dextran (Fig. 1B). Inoculation of PSaV together with 200 μM GCDCA increased diffusion by 24- and 14-fold for 4-kDa FITC-dextran and 70-kDa FITC-dextran, respectively (Fig. 1B). In addition, treatment of LLC-PK cell monolayers with GCDCA alone induced 6-fold and 2-fold increases in paracellular diffusion of 4-kDa FITC-dextran and 70-kDa FITC-dextran, respectively (Fig. 1B). Binding of PSaV VLPs showed a pattern similar to that of PSaV particles, leading to 20-fold and 10-fold increases in the diffusion of 4-kDa FITC-dextran and 70-kDa FITC-dextran, respectively (Fig. 1B). Taken together, these results indicate that PSaV infection induced dissociation of TJs, leading to an increase in paracellular permeability.

To further examine whether early disruption of TJs by PSaV influenced their fence functions, we analyzed the distribution of a lipid (Bodify FL-C12-sphingomyelin–bovine serum albumin [BSA]) in LLC-PK monolayers grown on transwell filters. The z-section presented in Fig. 1C shows that the lipid was confined in the apical domains of control cells and that no lateral staining was detected. However, incubation of cells with purified PSaV particles or VLPs for 1 h or control EGTA for 10 min resulted in the diffusion of the Bodify FL-C12-sphingomyelin–BSA complex across TJs and stained the lateral membranes (Fig. 1C). We also checked the distribution of the basolateral membrane marker Na^+^K^+^-ATPase after incubation with PSaV particles or VLPs. Mock-infected cells without any treatment showed clear basolateral distribution of Na^+^K^+^-ATPase (Fig. 1C). However, inoculation of LLC-PK with purified PSaV particles or VLPs or treatment with EGTA resulted in translocation of Na^+^K^+^-ATPase to the apical surface (Fig. 1C). These results further confirm that PSaV infection disrupts the TJs of cells.

### Occludin is internalized in response to PSaV infection.

**FIG 2:**
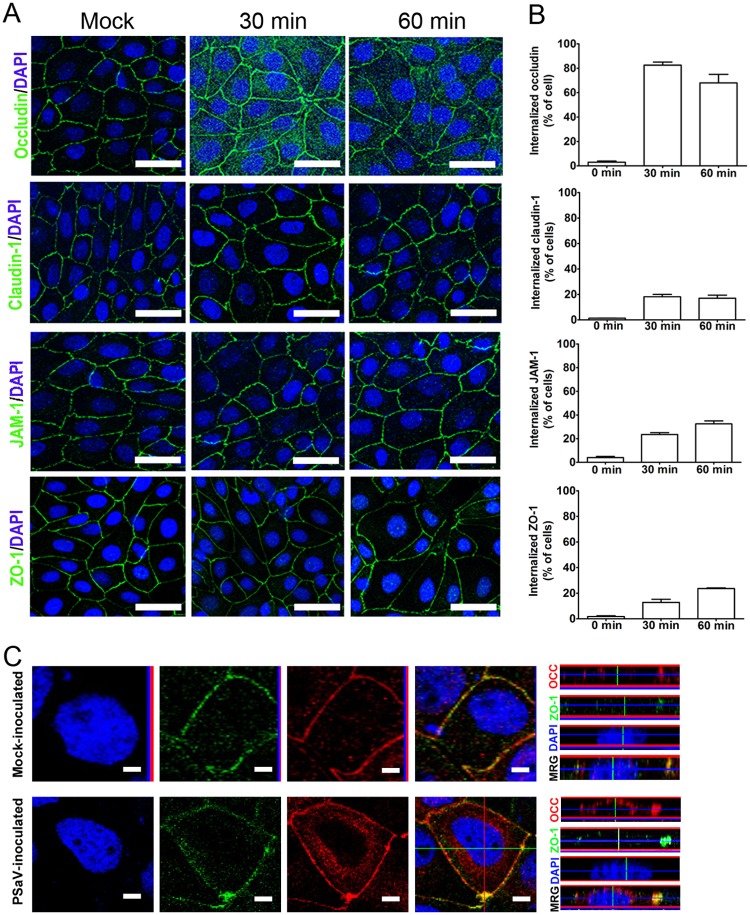
Occludin is internalized in response to PSaV infection. (A) LLC-PK monolayers infected or not with PSaV strain Cowden (MOI = 50) in the presence or absence of 200 μM GCDCA bile acid were incubated for 30 min or 60 min; fixed; and stained for occludin, claudin-1, JAM-1, and ZO-1 using each corresponding antibody. Scale bars, 50 μm. (B) Quantification of the internalization of occludin, claudin-1, JAM-1, and ZO-1 under the conditions for panel A was determined by counting cells containing each TJ protein in the cytoplasm and expressed as a percentage of the total cell numbers. The error bars indicate standard errors of the mean. (C) Confluent LLC-PK monolayers infected or not with PSaV (MOI = 50) in the presence or absence of 200 μM GCDCA bile acid were fixed and stained for occludin and ZO-1 at 30 min p.i. The cells were observed by x-sectioning (left) or z-sectioning (right) under a confocal microscope. Scale bars, 10 μm. All experiments were performed in triplicate, and representative images are shown in panels A and C.

During basolateral entry of viruses, including rotavirus, coxsackievirus B, and PEDV, TJ proteins have been observed to relocalize from the cell surface to the cytoplasm (5, 17, 18). Therefore, we explored the cellular distribution of TJ-associated proteins following PSaV infection. Confocal microscopy (CM) observation showed the sharp ring-like structures of the three transmembrane proteins occludin, claudin-1, and JAM-1, as well as their submembranous scaffolding protein, ZO-1, in control monolayers (Fig. 2A). However, among these proteins, intracytoplasmic translocation of occludin was more pronounced than that of claudin-1, JAM-1, or ZO-1 at early time points (30 and 60 min p.i. with PSaV) (Fig. 2A and B). This result was confirmed by localization of occludin during PSaV infection using z-stacking CM. Pronounced accumulation of occludin in the cytoplasm was evident, whereas ZO-1 remained in the paracellular junction area during early PSaV infection (Fig. 2C). These data indicate that PSaV infection promotes the internalization of occludin, which was more conspicuous than that of the other TJ-associated proteins investigated.

### Occludin functions as a coreceptor for PSaV.

**FIG 3:**
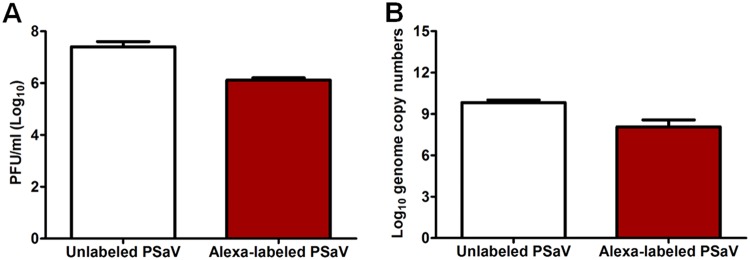
Mean numbers of viable virions were determined by plaque and quantitative real-time PCR assays before and afterAF594 labeling. (A and B) Purified PSaV strain Cowden (MOI = 50) was either labeled with AF594 or left unlabeled, and the virus titer was checked by plaque (A) and quantitative real-time PCR (B) assays as described in Materials and Methods. The error bars indicate standard errors of the mean.

**FIG 4:**
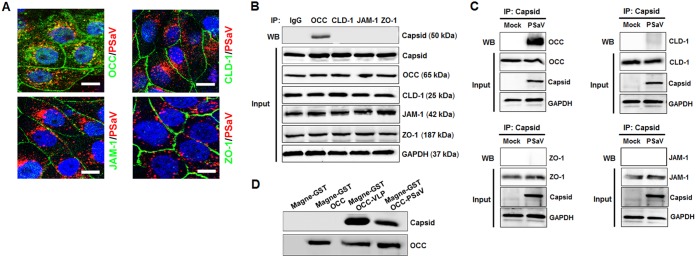
PSaV colocalizes and coprecipitates with occludin. (A) LLC-PK cells were inoculated with Alexa Fluor 594-labeled PSaV strain Cowden (4 × 10^2^ particles/cells) for 1 h on ice and shifted to 37°C for 30 min. The cells were fixed and incubated with antibodies specific for occludin (OCC), claudin-1 (CLD-1), JAM-1, or ZO-1. After washing twice with PBS-FBS, the cells were incubated with FITC-conjugated secondary antibodies at 20°C for 1 h. The cells were mounted with SlowFade Gold antifade reagent containing 1× DAPI solution for staining of nuclei. The slides were analyzed immediately by x-sectioning under a confocal microscope. One representative set of results is shown. The scale bars represent 20 μm. (B) The lysates of PSaV-infected LLC-PK cells were immunoprecipitated with antibodies specific for OCC, JAM-1, ZO-1, or CLD-1. As a mock control, immunoglobulin G was used for immunoprecipitation (IP) of the virus-infected cell lysates. The coimmunoprecipitated proteins were analyzed by Western blotting to detect OCC, JAM-1, ZO-1, or CLD-1 using the relevant antibody. To confirm the presence of each target protein in the cell lysates (input), the lysates of PSaV-infected LLC-PK cells were directly analyzed by Western blotting using the relevant antibody. (C) The lysates of PSaV-infected LLC-PK cells were immunoprecipitated with antibodies specific for PSaV capsid protein. As a mock control, mock-infected cell lysates were used for immunoprecipitation. The coimmunoprecipitated proteins were analyzed by Western blotting to detect occludin, JAM-1, ZO-1, or CLD-1 using the relevant antibody. To confirm the presence of each target protein and PSaV capsid protein in the cell lysates (input), the lysates of PSaV-infected LLC-PK cells were directly analyzed by Western blotting using the relevant antibody. (D) Soluble-form GST-tagged occludin (1 μg/ml) was mixed with PSaV particles (MOI = 50) or PSaV VLPs (4 μg/ml). Each complex was pulled down with MagneGST beads and then evaluated by Western blotting using antibody specific for PSaV capsid protein. The input occludin was also quantified by Western blot analysis. As a mock control, solution without PSaV particles, PSaV VLPs, and soluble-form GST-tagged occludin was pulled down with MagneGST beads and then evaluated by Western blotting using antibody specific for PSaV capsid protein or occludin. All experiments were performed in triplicate, and representative images from each group are presented.

To explore further whether PSaVs enter cells with the involvement of TJ proteins, we first examined the colocalization of PSaVs with TJ proteins. We first checked whether Alexa Fluor 594 (AF594)-labeled PSaV particles were infectious using plaque and quantitative real-time PCR (qPCR) assays. AF594-labeled PSaV particles were shown to be infectious, though their infectivity was 10-fold less than that of the purified nonlabeled PSaV particles (Fig. 3). Having established the infectivity of AF594-labeled PSaV particles, LLC-PK cells were infected with AF594-labeled PSaV and then examined by CM. As shown in Fig. 4A, AF594-labeled PSaV particles showed extensive colocalization with occludin. Some PSaV-free occludins were also internalized into the cytoplasm. No prominent interaction between PSaV particles and other major TJ transmembrane proteins, such as claudin-1, JAM-1, or the cytoplasmic scaffolding protein ZO-1, were visible by CM (Fig. 4A).

We next investigated whether PSaVs interact directly with TJ molecules as coreceptors. To assess this, LLC-PK cells were either mock infected or infected with the PSaV strain Cowden, and cell lysates were immunoprecipitated using antibodies specific for occludin, claudin-1, JAM-1, ZO-1, or PSaV capsid protein. The antibody specific for occludin significantly precipitated PSaV in comparison to precipitation by the antibodies specific to the other TJ molecules tested (Fig. 4B). Consistent with this result, antibody against PSaV capsid protein significantly precipitated occludin, but not claudin-1, JAM-1, and ZO-1 (Fig. 4C). To confirm the direct interaction between PSaV and occludin, we performed a pulldown assay using free glutathione *S*-transferase (GST)-tagged occludin and purified PSaV particles or PSaV VLPs. As shown in Fig. 4D, PSaV particles and VLPs were precipitated by GST-tagged occludin. The results of the colocalization experiment and the observed direct interaction between PSaV and occludin suggest that PSaV strain Cowden uses occludin as a coreceptor.

**FIG 5:**
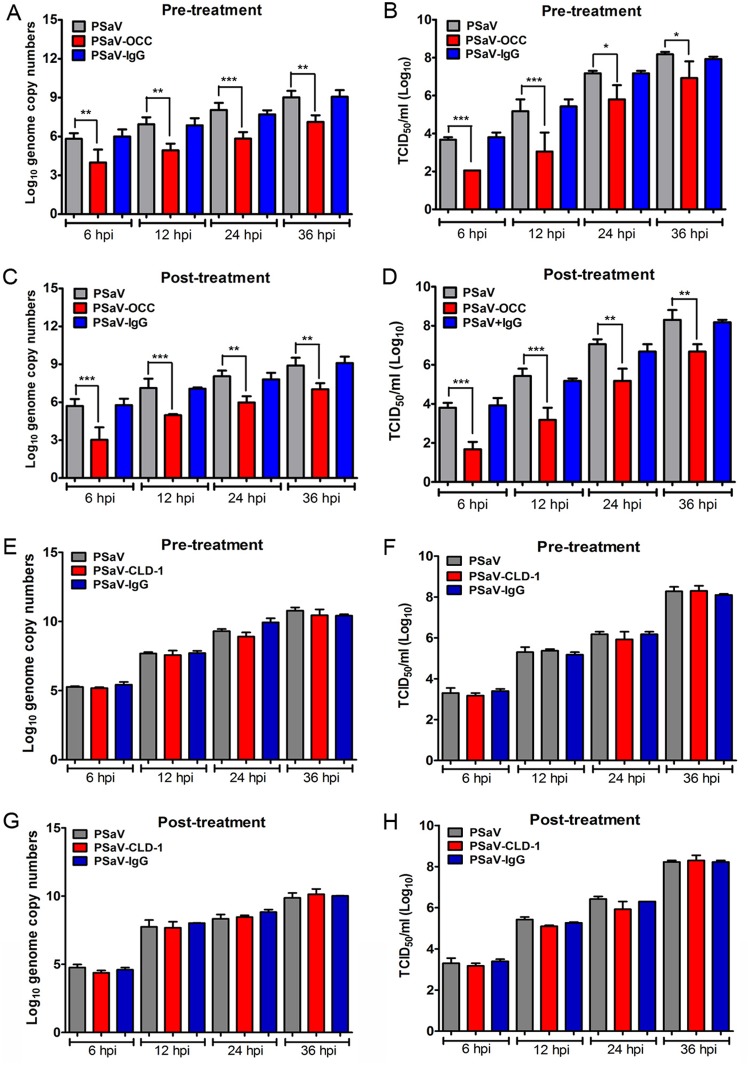
Occludin functions as a coreceptor for PSaV. LLC-PK cells were incubated with antibody against OCC, CLD-1, or a control IgG for 1 h at 4°C before (A, B, E, and F) or after (C, D, G, and H) attachment of PSaV strain Cowden (MOI = 50). After washing the cells with cold PBS, infection was allowed to proceed, and the cells were harvested at the indicated time points. Quantitative real-time PCR (A, C, E, and G) and TCID_50_ analyses (B, D, F, and H) were used to evaluate PSaV genome copy numbers and titers, respectively. All experiments were performed in triplicate. The data are presented as means and standard errors of the mean. *, *P* < 0.05; **, *P* < 0.001; ***, *P* < 0.0001.

To further test whether occludin functions as a coreceptor for PSaV, we treated cells with occludin- or claudin-1-specific monoclonal antibodies (MAbs) before virus inoculation or at various time points after virus adsorption to the cells. In cells treated with a MAb specific for extracellular loops of occludin, whether pre- or post-virus inoculation, PSaV genome copy numbers and PSaV titers were significantly decreased compared to those of mock-treated (without antibody treatment), PSaV-inoculated cells (Fig. 5A to D). However, pre- or posttreatment with MAb against claudin-1 had no significant effect on PSaV replication (Fig. 5E to H). Similar to what was observed during rotavirus entry (10), these results strongly suggest that occludin is required by PSaV at a postattachment step. Taken together, our data indicate that occludin functions as a coreceptor for PSaV.

### PSaV uses claudin as an entry factor.

**FIG 6:**
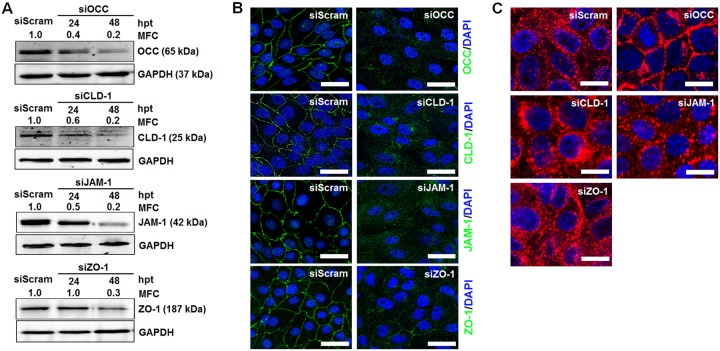
siRNAs against occludin and claudin-1 block PSaV entry. (A) LLC-PK cells at 70 to 80% confluence were transfected with 100 pmol of scrambled siRNA or 4 individual pooled siRNAs against OCC, CLD-1, JAM-1, or ZO-1. Cells were harvested 24 or 48 hpt, and Western blot analyses were performed to determine the downregulation of each target gene. GAPDH was used as a loading control. The mean fold change (MFC) was calculated using GelQuant.NET software (BiochemLab Solutions) by comparing the relative density of siRNA-treated samples to that of siScram-treated samples. (B) Monolayers of LLC-PK cells in 8-well chamber slides were transfected with siScram or siRNAs against OCC, CLD-1, JAM-1, or ZO-1 and stained with the relevant antibodies. The cells were mounted with SlowFade Gold antifade reagent containing 1× DAPI solution for staining of nuclei. The slides were analyzed immediately by x-sectioning under a confocal microscope. Scale bars, 20 μm. (C) Confluent LLC-PK cells in 8-well chamber slides were infected with Alexa Fluor 594-labeled PSaV strain Cowden (400 particles per cell) 48 h after transfection with scrambled, occludin (siOCC), claudin-1 (siCLD-1), JAM-1 (siJAM-1), or ZO-1 (siZO-1) siRNA and incubated for 30 min at 4°C in the presence of 200 μM GCDCA. Entry of Alexa Fluor 594-labeled PSaV particles commenced with a shift in the temperature to 37°C and incubation for 30 min. The cells were mounted with SlowFade Gold antifade reagent containing 1× DAPI solution for staining of nuclei. The slides were analyzed immediately by x-sectioning under a confocal microscope. Scale bars, 20 μm. All experiments were performed in triplicate, and representative results are shown.

**FIG 7:**
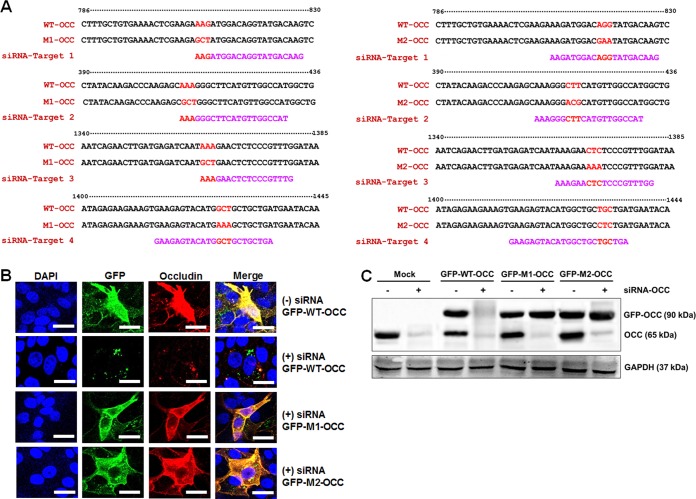
Determining off-target effects of siRNA against occludin. (A) Nucleotide sequences of wild-type (GFP-WT-OCC) and mutant (GFP-M1-OCC and GFP-M2-OCC) occludins and each siRNA. Using the plasmid encoding wild-type GFP-tagged occludin at the 5′ end (GFP-WT-OCC) and two mutant plasmids (GFP-M1-OCC and GFP-M2-OCC) containing four silent-mutation sites, each siRNA was generated by site-directed mutagenesis using primer pairs containing mutation sites as described in Materials and Methods. The red letters indicate the locations of mutation sites, and magenta letters indicate the unchanged siRNA sequence. (B and C) LLC-PK cells grown at 70 to 80% confluence on 8-well chamber slides were transfected with GFP-WT-OCC or GFP-M1-OCC and GFP-M2-OCC mutant plasmids. At 4 hpt, individually with each plasmid, the cells were then transfected with 4 individual pooled siRNAs against occludin, and knockdown effects by occludin siRNAs were examined by confocal microscopy (B) and Western blot analyses (C) using antibody specific for occludin as described in Materials and Methods. GAPDH was used as a loading control in panel C. The scale bars in panel B represent 20 μm.

To further examine whether inhibition of TJ molecules can impair PSaV entry, we silenced the expression of occludin, claudin-1, JAM-1, and ZO-1 in LLC-PK cells. Cells were transfected with pooled small interfering RNAs (siRNAs) targeting four individual sites in each gene, helping to reduce off-target effects. Subsequent protein expression was examined by Western blot analysis and CM. Expression of occludin, claudin-1, JAM-1, and ZO-1 was significantly reduced at 48 h posttransfection (hpt) compared to protein expression at 24 hpt (Fig. 6A). This was further confirmed by CM (Fig. 6B). We next investigated the knockdown effects of these TJ proteins on PSaV entry. As expected, silencing of occludin expression significantly inhibited the entry of AF594-labeled PSaV particles into the cells, whereas transfection with scrambled siRNA (siScram) allowed the entry of AF594-labeled PSaV particles into the cytoplasm (Fig. 6C). Unexpectedly, knockdown of claudin-1 had a moderate inhibitory effect on PSaV entry into cells (Fig. 6C). In addition, siRNA knockdown of JAM-1 or ZO-1 had no effect on PSaV entry (Fig. 6C). To assess whether off-target effects of siRNAs contributed to the results described above in occludin-silenced cells, we constructed a plasmid (GFP-WT-OCC) containing a green fluorescent protein (GFP)-tagged wild-type occludin gene and two mutant plasmids (GFP-M1-OCC and GFP-M2-OCC) containing four silent-mutation sites corresponding to the four pooled siRNAs. The occludin-siRNA target sequences and mutation sites are shown in Fig. 7A. Cells harboring GFP-WT-OCC were sensitive to transfection with the pooled siRNAs targeting occludin (Fig. 7B and C), while cells harboring GFP-M1-OCC or GFP-M2-OCC were insensitive to transfection with the pooled siRNAs (Fig. 7B and C). These results indicate that occludin is required by PSaV at a postattachment step and suggest that claudin-1 may function as an entry factor.

**FIG 8:**
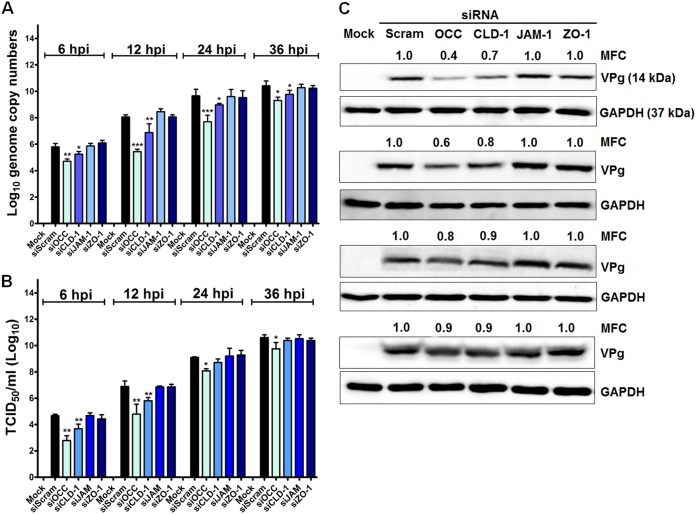
siRNAs against occludin and claudin-1 block PSaV infection. LLC-PK cells at 70 to 80% confluence grown in 6-well plates were infected with PSaV strain Cowden (MOI = 50) 48 h after transfection with 100 pmol siScram, siOCC, siCLD-1, siJAM-1, or siZO-1 and incubated in the presence of 200 μM GCDCA for the indicated times. (A) The PSaV genome copy numbers at each harvest time point were determined by quantitative real-time PCR, and the inhibitory effect of each siRNA on PSaV genome replication was compared with that of scrambled-siRNA-transfected, PSaV-infected cells. (B) LLC-PK cells grown in 96-well plates were inoculated with 200 μl of 10-fold serial dilutions of virus-containing supernatant and incubated at 37°C for 6 days. Virus titers were determined by the Reed-Muench method and expressed as TCID_50_ per milliliter. The inhibitory effect of each siRNA on the PSaV titer was compared with that of scrambled-siRNA-transfected, PSaV-infected cells. (C) The cell lysates harvested at the indicated time points were analyzed by Western blotting using antibody specific for PSaV VPg protein. GAPDH was used as a loading control. The MFC was calculated using GelQuant.NET software by comparing the relative density of siRNA-treated samples to that of scrambled-siRNA-transfected samples. Triplicate experiments were performed, and the data in panels A and B are presented as means and standard errors of the mean. *, *P* < 0.05; **, *P* < 0.001; ***, *P* < 0.0001.

**FIG 9:**
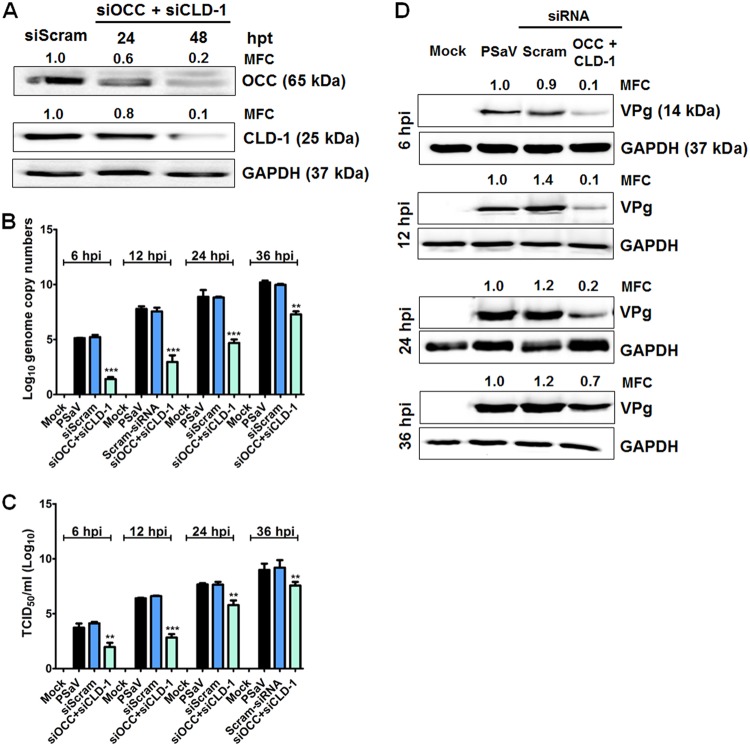
Synergistic effect of combined siRNA against occludin and claudin-1. (A) LLC-PK cells grown in 6-well plates at 70 to 80% confluence were transfected with combined siRNAs against occludin and claudin-1 or scrambled siRNA. At 48 hpt, cells were harvested, and knockdown of their target proteins was assessed by Western blot analysis. GAPDH was used as a loading control. The MFC was calculated using GelQuant.NET software by comparing the relative density of siRNA-treated samples to that of siScram-treated samples. (B to D) LLC-PK cells were transduced with combined siRNAs against occludin and claudin-1 for 48 h. Next, the cells were infected with PSaV strain Cowden (MOI = 50) in the presence of 200 μM GCDCA for the indicated times. (B) The PSaV genome copy numbers at the indicated time points were determined by quantitative real-time PCR, and the inhibitory effects of combined siRNAs against occludin and claudin-1 were compared with those of scrambled-siRNA-transfected and PSaV-infected cells. (C) LLC-PK cells grown on 96-well plates were infected with 200 μl of 10-fold serial dilutions of virus-containing supernatant and incubated at 37°C for 6 days. Virus titers were determined by the Reed-Muench method and expressed as TCID_50_ per milliliter. The inhibitory effect of each siRNA on the PSaV titer was compared with that of scrambled-siRNA-transfected, PSaV-infected cells. (D) The cell lysates harvested at the indicated time points were analyzed by Western blotting using antibody specific for PSaV VPg protein. GAPDH was used as a loading control. The MFC was calculated using GelQuant.NET software by comparing the relative density of siRNA-treated samples to that of scrambled-siRNA-transfected samples. Experiments were performed in triplicate, and the data are presented as means and standard errors of the mean. **, *P* < 0.001; ***, *P* < 0.0001.

To confirm the above-mentioned results, cells transfected with siRNAs against the TJ proteins were inoculated with PSaV particles and incubated for various lengths of time. A significant reduction in PSaV genome replication, PSaV VPg protein synthesis, and PSaV titers was observed in occludin knockdown cells from 6 h p.i. until 36 h p.i. (Fig. 8). Moreover, knockdown of claudin-1 also significantly decreased PSaV genome copy numbers, PSaV VPg protein synthesis, and PSaV titers (Fig. 8). However, silencing of JAM-1 and ZO-1 had no inhibitory effects on PSaV genome replication, PSaV VPg protein synthesis, or PSaV titers (Fig. 8). We further examined the effects of silencing of occludin and claudin-1 together on PSaV VPg protein synthesis and PSaV titers. Transfection of combined siRNAs against occludin and claudin-1 led to the downregulation of their target proteins, as shown by Western blot analysis (Fig. 9A). As expected, when siRNAs for occludin and claudin-1 were combined, a significant decrease in PSaV genome copy numbers, titers, and VPg protein synthesis were observed (Fig. 9B to D). Taken together, our results indicate that PSaV utilizes occludin as a coreceptor and that claudin-1 may serve as an entry factor.

### Occludin expression confers PSaV susceptibility on nonpermissive cells.

**FIG 10:**
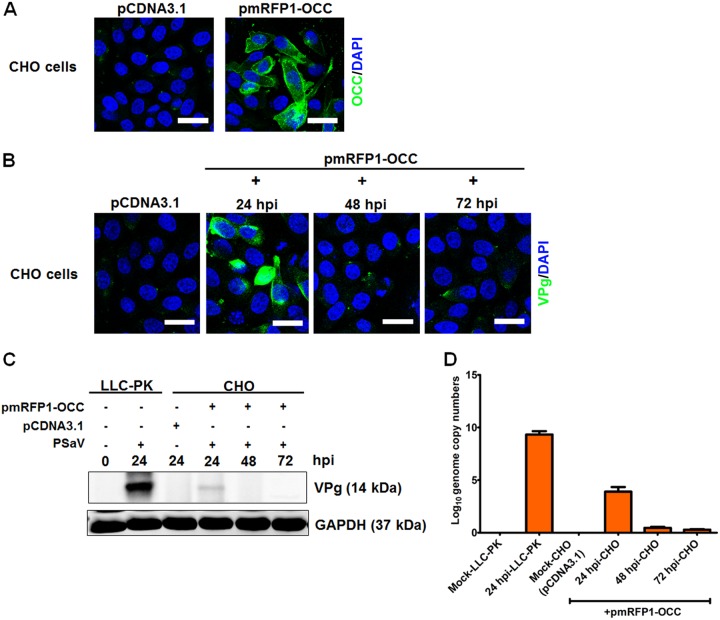
PSaV infection in nonpermissive cells expressing occludin. (A) CHO cells were transiently transduced with 1 μg of pCDNA3.1 or pmRFP1-occludin (pmRFP1-OCC), and expression of occludin was checked by confocal microscopy using antibody specific for occludin. (B) CHO cells grown in 8-well chamber slides were transfected with pCDNA3.1 or pmRFP1-OCC plasmid and then incubated for 24 h. The cells were then infected with PSaV (MOI = 50) and incubated for the indicated times. The cells were washed, fixed with 4% paraformaldehyde, permeabilized with 0.01% Triton X-100, and then stained with antibody specific for PSaV VPg. The cells were mounted with SlowFade Gold antifade reagent containing 1× DAPI solution for staining of nuclei. The slides were analyzed immediately by x-sectioning under a confocal microscope. (C) CHO cells grown in 6-well plates at 70 to 80% confluence were transfected with 1 μg pmRFP1-OCC. At 24 h posttransfection, the cells were infected with PSaV strain Cowden (MOI = 50) and incubated for the indicated times. LLC-PK monolayers grown in 6-well plates were inoculated with PSaV strain Cowden (MOI = 50) and incubated for 24 h. The cell lysates harvested at the specified time points were analyzed by Western blotting using antibody specific for PSaV VPg protein. GAPDH was used as a loading control. The MFC was calculated using GelQuant.NET software by comparing the relative density of siRNA-treated samples to that of siScram-treated samples. Experiments were performed in triplicate, and representative images are presented in panels A to C. (D) CHO cells grown in 6-well plates were transfected and incubated with pCDNA3.1 or pmRPF1-OCC plasmid for 24 h and then infected with PSaV strain Cowden (MOI = 50). The CHO cells were harvested at 24, 48, and 72 h p.i. LLC-PK cells cultured in 6-well plates were mock infected or infected with PSaV strain Cowden (MOI = 50) and harvested at 24 h p.i. All the samples were processed for quantitative real-time PCR to determe viral genome copy numbers as described in Materials and Methods. Data are presented as means and standard errors of the mean for triplicate experiments.

To investigate if occludin expression could facilitate PSaV infection, Chinese hamster ovary (CHO) cells, which do not express occludin (51), were transfected with an occludin-expressing plasmid, pmRFP1-OCC. CHO cells transfected with pmRFP-OCC expressed occludin, whereas CHO cells transfected with a control plasmid, pCDNA3.1, did not (Fig. 10A). CHO cells transfected with pCDNA3.1 or pmRFP-OCC were infected with PSaV particles at a multiplicity of infection (MOI) of 50 in the presence of 200 μM GCDCA and harvested at 24, 48, and 72 h p.i. The samples were subjected to Western blotting, qPCR, and CM analyses. The CHO cells transfected with pCDNA3.1 did not allow virus replication (Fig. 10B to D). However, PSaV replication was evident in pmRFP-OCC-transfected CHO cells at 24 h p.i. but was markedly decreased by 48 h p.i. (Fig. 10B). The viral protein VPg and the genome were detected at 24 h p.i. (Fig. 10B and C) but were significantly decreased by 48 h p.i. (Fig. 10B and C). Compared with LLC-PK cells infected with PSaV, VPg expression and genome copy numbers were very low in pmRFP-OCC-transfected CHO cells (Fig. 10B and C). This may have been due to missing factors needed to sustain PSaV replication. These results suggest that ectopic expression of occludin in nonpermissive cells renders them susceptible to PSaV replication.

### PSaV and occludin travel to late endosomes together via Rab5- and Rab7-dependent trafficking.

**FIG 11:**
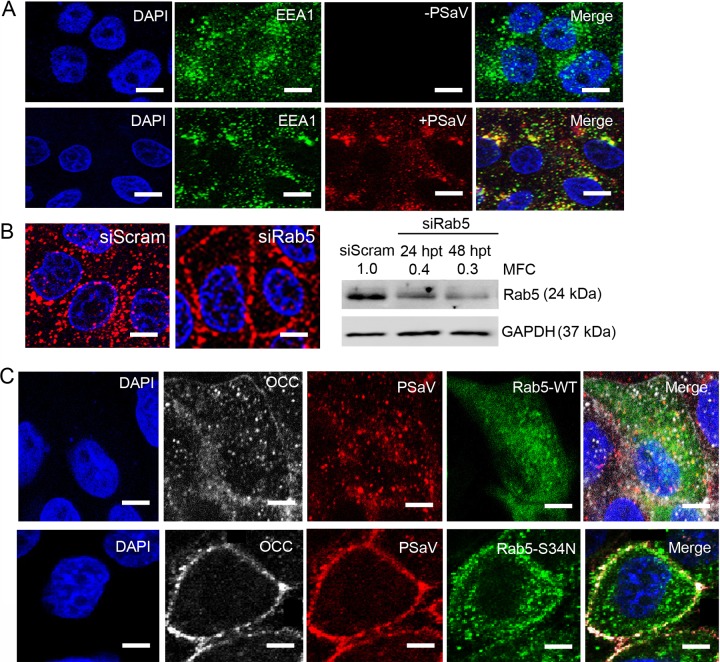
Coentry of occludin and PSaV depends on Rab5. (A) Confluent LLC-PK cells grown in 8-well chamber slides were inoculated with Alexa Fluor 594-labeled PSaV (MOI = 50), incubated for 1 h at 4°C, and then incubated for another 30 min at 37°C. Confocal microscopy was performed to detect the colocalization of Alexa Fluor 594-labeled PSaV and the early endosome marker EEA1. Scale bars, 50 μm. (B) LLC-PK cells at 70 to 80% confluence were infected with Alexa 594-labeled PSaV (MOI = 50) 48 h after transfection with scrambled siRNA or siRNAs against Rab5 and then incubated at 37°C for 30 min. The knockdown effects on Rab5 protein by scrambled siRNA or siRNAs against Rab5 was evaluated by Western blot analysis. GAPDH was used as a loading control. The MFC was calculated using GelQuant.NET software by comparing the relative density of siRNA-treated samples to that of scrambled-siRNA-transfected samples. Scale bars, 50 μm. (C) LLC-PK cells transfected with either wild-type EGFP-Rab5 or a dominant-negative mutant (S34N) plasmid were infected with Alexa Fluor 594-labeled PSaV (MOI = 50) and stained for occludin (white) at 30 min postinoculation. The cells were mounted with SlowFade Gold antifade reagent containing 1× DAPI solution for staining of nuclei. The slides were analyzed immediately by x-sectioning under a confocal microscope. Scale bars, 20 μm. All experiments were performed in triplicate, and representative images are presented.

**FIG 12:**
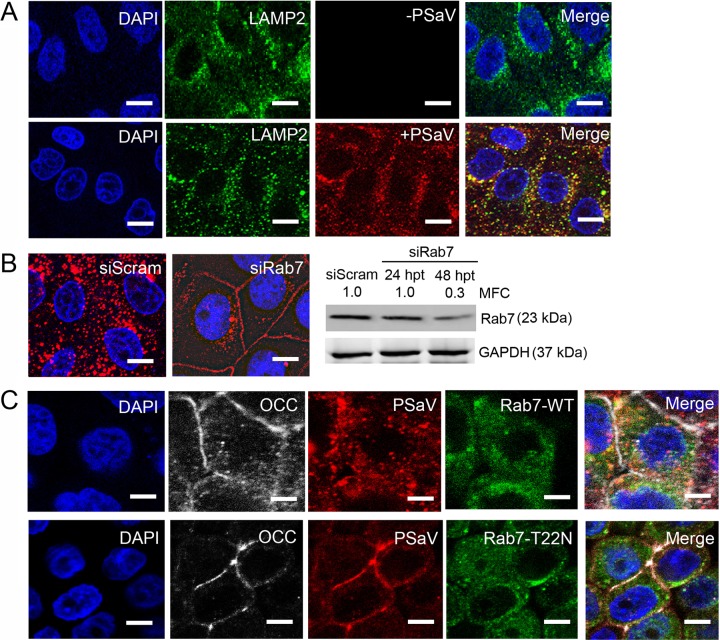
Coentry of occludin and PSaV depends on Rab7. (A) Confluent LLC-PK cells grown in 8-well chamber slides were inoculated with Alexa Fluor 594-labeled PSaV (MOI = 50), incubated for 1 h at 4°C, and then incubated for another 60 min at 37°C. Colocalization of Alexa 594-labeled PSaV and the late endosome marker LAMP2 was evaluated by confocal microscopy. Scale bars, 50 μm. (B) LLC-PK cells at 70 to 80% confluence were infected with Alexa Fluor 594-labeled PSaV (MOI = 50) 48 h after transfection with scrambled siRNA or siRNA against Rab7 and then incubated at 37°C for 30 min. The knockdown effects on Rab7 protein by scrambled siRNA or siRNAs against Rab7 were evaluated by Western blot analysis. GAPDH was used as a loading control. The MFC was calculated using GelQuant.NET software by comparing the relative density of siRNA-treated samples to that of scrambled-siRNA-transfected samples. Scale bars, 50 μm. (C) LLC-PK cells transfected with either wild-type-form EGFP-Rab7 or a dominant-negative mutant (T22N) were infected with Alexa Fluor 594-labeled PSaV particles (MOI = 50) and stained for occludin (white) at 60 min postinoculation. The cells were mounted with SlowFade Gold antifade reagent containing 1× DAPI solution for staining of nuclei. The slides were analyzed immediately by x-sectioning under a confocal microscope. Scale bars, 20 μm. All experiments were performed in triplicate, and representative images are shown.

Small GTPases in the Rab family regulate membrane-trafficking events, including endocytosis. Rab5 is a key regulator controlling the internalization of vesicles and fusion with early endosomes (52), whereas Rab7 is primarily associated with late endosomal maturation and endosome-lysosome fusion (53). These characteristics can be used to differentiate between endosomal compartments: early endosomes contain Rab5 and its effector, EEA1, whereas late endosomes contain Rab7 and its effector, LAMP2 (54). PSaV strain Cowden is known to travel from early to late endosomes prior to PSaV uncoating and genome release into the cytoplasm (55, 56). Therefore, we examined trafficking of PSaV from early to late endosomes prior to determining whether PSaV and occludin travel in a complex to these endosomal compartments. Consistent with previous reports (55, 56), most AF594-labeled PSaV particles colocalized with the early endosome marker EEA1 at 30 min p.i. and with the late endosome marker LAMP2 at 60 min p.i. (Fig. 11A and 12A), confirming the journey of PSaV from early to late endosomes (55, 56).

We next examined whether silencing of Rab5 or Rab7 blocked travel of AF594-labeled PSaV particles, colocalized with occludin, to the different endosomal compartments. LLC-PK cells transfected for 48 h with siRNAs against either Rab5 or Rab7 or with scrambled siRNA were inoculated with AF594-labeled PSaV particles. Colocalization of AF594-labeled PSaV particles and occludin was analyzed at 30 min p.i. in the Rab5 knockdown cells or at 60 min p.i. in the Rab7 knockdown cells. Transfection of scrambled siRNA had no inhibitory effects on the movement of colocalized PSaV-occludin complexes into the perinuclear area of the cytoplasm, whereas transfection of either Rab5 or Rab7 siRNA in the cells resulted in trapped PSaV particles at the periphery of the cytoplasm (Fig. 11B and 12B). To confirm the transit of PSaV-occludin complexes from early to late endosomes, LLC-PK cells were transfected with wild-type enhanced green fluorescent protein (EGFP)-Rab5 (EGFP-Rab5WT) or EGFP-Rab7 (EGFP-Rab7WT) plasmids or plasmids containing dominant-negative mutants of EGFP-Rab5 (EGFP-Rab5S34N) or EGFP-Rab7 (EGFP-Rab7T22N) and then inoculated with AF594-labeled PSaV particles. PSaV particles, colocalized with occludin, accumulated particularly in the perinuclear areas of the cells transfected with either Rab5- or Rab7-WT, whereas transfection of the dominant-negative mutant S34N-Rab5 or T22N-Rab7 partially blocked entry of AF594-labeled PSaV particles colocalized with occludin, which remained at the periphery of the cytoplasm (Fig. 11C and 12C). Taken together, these data indicate that PSaV and occludin travel as complexes from early to late endosomes via Rab5- and Rab7-dependent trafficking.

## DISCUSSION

The initial event in the virus life cycle is binding of the virus particles to specific cell surface receptors, enabling their entry into cells and the initiation of pathology (4, 35). Therefore, interactions between viruses and host cell receptors are a crucial step affecting viral tropism, pathogenesis, and host range (35, 57). Accumulating evidence indicates that viruses enter cells through a complex multistep process in which many viruses, such as rotavirus and HCV, require more than one receptor for virus entry and infection (45, 58, 59). Members of at least nine different virus families have been reported to use TJ proteins as receptors (4, 45). Although cell surface carbohydrate moieties, either terminal SAs or HBGAs, are used by some caliciviruses as attachment factors (36, 37), cell surface proteins are also utilized as receptors by MNoV, FCV, and Hom-1 vesivirus, with the last two viruses recognizing a TJ protein, JAM-1, as a receptor (40–42, 44, 60). The usage of α2,3- and α2,6-linked SAs on O-linked glycoproteins as attachment factors for PSaV entry and infection was previously described (36). In the present study, we found that PSaV and occludin interact directly and enter cells as a complex, as demonstrated by treatment of cells with specific siRNAs or MAbs. These data indicate that, similar to FCV and Hom-1 vesivirus, PSaV utilizes a TJ protein, occludin, as a coreceptor (44, 60). Given the previous results (36), we conclude that PSaV first attaches to SAs on the apical cell surface, dissociates TJs, moves into the TJs, and then binds to occludin for successful entry and infection.

Cell-cell adhesion is safeguarded and sealed by the apical-junctional complex, composed of TJs, adherent junctions, and desmosomes (8, 61). Therefore, viruses utilizing apical-junctional complex molecules as receptors have devised specific strategies to disrupt the apical-junctional complex as a part of their cellular pathogenesis, resulting in successful entry and later clinical consequences, such as diarrhea in the case of sapovirus infections (4, 5, 8, 18). In this context, we hypothesized that PSaVs disrupt and then open TJs prior to attachment to occludin. In the present study, we showed that early infection of PSaV induced decreased TER in conjunction with increased paracellular permeability. Moreover, alterations in the TJ fence function in response to PSaV infection were observed, as shown by lateral diffusion of the lipid probe, Bodify FL-C12-sphingomyelin–BSA complex, administered apically. The rotavirus surface protein VP8* modulates the gate and fence functions of TJs in epithelial cells, and the VP4 surface protein of some rotavirus strains defines the requirement for JAM-A as a basolateral coreceptor (5, 10). Similar to the mechanism of basolateral entry by rotaviruses, we have shown that PSaV can dissociate TJs, exposing occludin, which is sequestered in the apical-junctional complexes at the lateral surfaces of cells. PSaV then hijacks occludin as a coreceptor for entry and infection. In addition, a relatively large proportion of intracellular occludin did not colocalize with PSaV particles. Although the reason for this is not known, binding of PSaV particles to attachment receptors may activate signaling pathways that facilitate internalization of virus-free occludin into the cytoplasm. Further studies are required to examine whether PSaV induces activation of such a signaling pathway(s).

In addition to the role of apical-junctional complexes as receptors or coreceptors, they can also be used by many viruses as entry factors (4). In the present study, claudin-1, JAM-1, and ZO-1 were less extensively internalized into cells in response to PSaV infection and did not colocalize with PSaV in the cytoplasm. Moreover, direct interaction between these proteins and PSaV was not evident by immunoprecipitation assay. However, transfection of siRNA targeting claudin-1 partially blocked PSaV entry, as well as significantly reducing viral genome copy numbers, protein synthesis, and titers. Similar findings have been observed in studies of the entry processes of some viruses, including rotavirus, PED coronavirus, and coxsackie B virus. In these studies, TJ proteins, such as claudin-1, were found to serve as scaffolds that recruit and anchor signaling or regulatory molecules to the vicinity of virus entry (10, 17, 18). Taken together, our results suggest that claudin-1 serves as a scaffold at the site of PSaV entry. The functional roles of other claudins as potential entry receptors or entry factors for PSaV warrant further research.

The PSaV strain Cowden is the only cultivable member of the genus *Sapovirus* and can grow in a porcine kidney cell line in the presence of intestinal contents or bile acid (31). In characterizing the role of occludin in PSaV entry, the ectopic expression of occludin in CHO cells rendered them susceptible to infection. However, the replicative cycle of PSaV was not sustained in occludin-expressing CHO cells. This may be due to insufficient host cell machinery required for viral protein and RNA synthesis, as well as virion assembly. This finding was in contrast to results described for Hom-1 calicivirus, where transfection of human JAM-1 (hJAM-1) in CHO cells enabled successful replication of virus (44). Further studies are needed to find other cells suitable for investigation of PSaV infection and to discover the host machinery required for PSaV replication.

Inclusion of bile acid or intestinal content in the cell culture medium is an essential prerequisite for successful propagation of PSaV and some strains of human norovirus *in vitro* (31, 33, 34, 62). Bile acids are critical for PSaV genome escape from late endosomes into the cell cytoplasm to start viral replication (55). Interestingly, in the present study, the addition of the bile acid GCDCA decreased TER and increased paracellular permeability in LLC-PK cells, thereby aiding in the dissociation of TJs. This suggests that, in addition to aiding PSaV escape from late endosomes, bile acids can facilitate early interactions between PSaV and occludin through the dissociation of TJs. Bile acids have previously been reported to modulate intestinal permeability by autophosphorylation of the epithelial growth factor (EGF) receptor and dephosphorylation and rearrangement of occludin at TJs (63). Moreover, the role of bile acids in opening TJs is known to be mediated by *src* family kinases and is ameliorated by EGF treatment (63). A correlation between the presence of bile acid and TJ modification upon PSaV entry has not been fully investigated. Therefore, further studies are required to elucidate this particular function of bile acid during PSaV entry.

Upon internalization, most viruses travel to different endosomal compartments for subsequent uncoating and cytoplasmic invasion (54). Of the various small GTPases present on endosomes, Rab5 and Rab7 are critical for the function of early endosomes and late endosomes, respectively (64, 65). Consistent with the results of previous studies (55, 56), we found that PSaV particles progressed from EEA1-positive early endosomes to LAMP2-positive late endosomes very early in infection and that this trafficking was decreased by siRNAs specific for the early endosome marker Rab5 or the late endosome marker Rab7. Interestingly, inhibition of Rab5 or Rab7 by transfection of siRNAs diminished the coentry of PSaV and occludin into the cytoplasm. These results were confirmed by transfection of plasmids expressing dominant-negative mutants of Rab5 (S34N) or Rab7 (T22N), which also inhibited trafficking of PSaV particles in complex with occludin from early to late endosomes. The direct interaction between PSaV and occludin, as well as entry of PSaV and occludin as complexes into the cytoplasm, suggested that these complexes travel from early to late endosomes. This result was similar to those of previous studies, which showed that, regardless of the usage of TJ proteins as receptors, TJ proteins internalized into cells during virus entry, or during constitutive trafficking, could be transported to their endosomal compartments (18, 50, 66).

In summary, we found that PSaV induces early dissociation of TJs, before binding to occludin as a coreceptor, and that PSaV-occludin complexes then travel to late endosomes, mediated by Rab5- and Rab7-dependent trafficking. This study contributes to understanding of cell entry by sapovirus and other caliciviruses and of potential targets for efficient and affordable antisapovirus therapies.

## MATERIALS AND METHODS

### Cell lines and virus.

Porcine kidney LLC-PK cells were routinely grown in Eagle’s minimal essential medium (MEM) supplemented with 10% fetal bovine serum (FBS) and 1% penicillin-streptomycin at 37°C in a 5% CO_2_ atmosphere. CHO cells were grown in Dulbecco’s modified Eagle medium (DMEM) supplemented with 10% FBS and 1% penicillin-streptomycin at 37°C in a 5% CO_2_ atmosphere. Spodoptera frugiperda ovarian cells (Sf9 cells) purchased from Gibco were cultured at 27°C in SF-900 II SFM medium containing 10% FBS, 100 U/ml penicillin, 100 μg/ml streptomycin, a lipid medium supplement, and 0.1% pluronic acid solution (Sigma-Aldrich, St. Louis, MO, USA). PSaV strain Cowden was generated from a full-length infectious cDNA clone, pCV4A (a kind gift from K. O. Chang, Kansas State University, Manhattan, KS, USA), and cultured in LLC-PK cells supplemented with 200 μM GCDCA (Sigma-Aldrich), 2.5% FBS, and 1% penicillin-streptomycin (33, 62). To obtain a high virus titer and to remove cellular factors, PSaVs were purified by cesium chloride (CsCl) gradient centrifugation as described below (57). The viral titer was determined by 50% tissue culture infective dose (TCID_50_) assay as described previously (51).

### Antibodies and reagents.

MAbs against claudin-1, JAM-1, and ZO-1 were purchased from Invitrogen (Life Technologies). Rabbit polyclonal antibodies against Rab5, Rab7, and LAMP2 were obtained from Abcam (Cambridge, United Kingdom). A MAb against EEA1 was purchased from BD Transduction Laboratories. MAbs against occludin and Na^+^K^+^-ATPase and rabbit anti-glyceraldehyde 3-phosphate dehydrogenase (GAPDH) (FL–335) polyclonal antibody were obtained from Santa Cruz. Goat immunoglobulin against rabbit IgG was purchased from Cell Signaling. Rabbit polyclonal antibodies against PSaV capsid or VPg were generated by immunization of a New Zealand White rabbit with purified capsid protein and VPg (36). The secondary antibodies used were AF488- or AF647-conjugated goat anti-rabbit or anti-mouse immunoglobulin (Molecular Probes). Horseradish peroxidase (HRP)-conjugated goat anti-rabbit and anti-mouse secondary antibodies were obtained from Santa Cruz. GST-tagged recombinant occludin protein was purchased from Abcam, MagneGST glutathione particles were obtained from Promega, AF594 succinimidyl ester was purchased from Molecular Probes, and SlowFade Gold antifade reagent with 4′,6-diamidino-2-phenylindole (DAPI) was obtained from Molecular Probes. Three individual pooled siRNAs specific for Rab5 or Rab7 and scrambled siRNA were purchased from Invitrogen, and four individual pooled ON-Target plus siRNAs against occludin, JAM-1, claudin-1, and ZO-1 were obtained from Dharmacon (Table 1). The pmRFP1-occludin plasmid was kindly provided by Jerrold R. Turner (University of Chicago). Plasmids encoding a wild-type Rab5 (EGFP-Rab5WT) or its dominant-negative mutant (EGFP-Rab5S34N) were kindly provided by Marino Zerial (Max Planck Institute of Molecular Cell Biology and Genetics, Dresden, Germany). Plasmids encoding wild-type Rab7 (EGFP-Rab7WT) and its dominant-negative mutant (EGFP-Rab7T22N) were generous gifts from Craig Roy (Yale University, New Haven, CT, USA).

**TABLE 1:** Sequences of siRNAs against each target gene and scrambled siRNA used in this study^*a*^

Target gene	Set	Orientation	Sequence (5′ to 3′)
Occludin	A	Sense	GAAGAAAGAUGGACAGGUA
		Antisense	UACCUGUCCAUTCTTTCTTC
	B	Sense	UUUGGGCTTCUTGTTGGCCUT
		Antisense	TAGGCCAACAAGAAGCCCAAA
	C	Sense	AUAAAGAACUCUCCCGUUU
		Antisense	AAACGGGAGAGUUCUUUAU
	D	Sense	GUACAUGGCUGCUGCUGAU
		Antisense	AUCAGCAGCAGCCAUGUAC
Claudin-1	A	Sense	GCAAUAGAAUCGUUCAAGA
		Antisense	UCUUGAACGAUUCUAUUGC
	B	Sense	GGAAAGACUACGUGUGACA
		Antisense	UGUCACACGUAGUCUUCCU
	C	Sense	CACCAAGGCCCUAUCCAAA
		Antisense	UUUGGAUAGGGCCUUGGUG
	D	Sense	GAGGAUGGCUGUCAUUGGG
		Antisense	CCCAAUGACAGCCAUCCUC
JAM-1	A	Sense	GGAUAGUGAUGCCUACGAA
		Antisense	UUCGUAGGCAUCACUAUCC
	B	Sense	CGAGUAAGAAGGUGAUUUA
		Antisense	UAAAUCACCUUCUUACUCG
	C	Sense	GGGAAGACACUGGGACAUA
		Antisense	UAUGUCCCAGUGUCUUCCC
	D	Sense	GGAAGGCGGCAACAGCUAU
		Antisense	AUAGCUGUUGUUGCCUUCC
ZO-1	A	Sense	GAGAAGAAGUGACCAUAUU
		Antisense	AAUAUGGUCACUUCUUCUC
	B	Sense	CUACACUGAUCAAGAACUA
		Antisense	UAGUUCUUGAUGAGUGUAG
	C	Sense	CGAGCGAUCUCAUAAACUU
		Antisense	AAGUUUAUCAGAUCGCUCG
	D	Sense	GAGGGUACUUUCCACGUUU
		Antisense	AAACGUGGAAAGUACCCUC
Rab5	A	Sense	GCAAGUCCUAACAUUGUAAtt
		Antisense	UUACAAUGUUAGGACUUGCtt
	B	Sense	CCAAAGAAUGAACCACAAAtt
		Antisense	UUUGUGGUUCAUUCUUUGGtt
	C	Sense	GUACCCGUAAUUUGUAACAtt
		Antisense	UGUUACAAAUUACGGGUACtt
Rab7	A	Sense	GGAAGACAUCACUCAUGAAtt
		Antisense	UUCAUGAGUGAUGUCUUCCtt
	B	Sense	CCAGUAUGUGAAUAAGAAAtt
		Antisense	UUUCUUAUUCACAUACUGGtt
	C	Sense	GCGUUCUGGUAUUUGAUGUtt
		Antisense	ACAUCAAAUACCAGAACGCtt
Scrambled	A		r(UUCUCCGAACGUGUCACGU)d(TT)

a Lowercase letters, hanging sequences. Restriction enzyme sites in the forward primer (F; atGGTACCagATGTCATCCAGGCCTCTTGAAAGT) (KpnI) and reverse primer (R; tcGGATCCaCTATGTTTTCTGTCTATCATAGTCTCCAACCATC) (BamHI) for occludin are underlined.

### Preparation of PSaV VLPs.

VLPs of PSaV strain Cowden were generated in baculovirus-infected Sf9 cells using the Bac-to-Bac baculovirus expression system (Invitrogen) according to the manufacturer’s instructions (67). Briefly, complete DNA encoding the major capsid protein VP1 and the minor capsid protein VP2 were amplified by reverse transcription (RT)-PCR with primers specific for the entire VP1 and VP2 regions (forward primer, 5′**-**ATG GAG GCG CCT GCC CC-3′; reverse primer, 5′**-**C ACT CAC AGC AAA GTG TGA TGA-3′) using RNA extracted from the supernatants of PSaV-infected cells. Subsequently, the amplified fragments were subcloned into a pFastBac1 donor plasmid. Recombinant baculovirus was generated by transformation of the recombinant pFastBac1 plasmid into Escherichia coli DH10Bac to produce recombinant bacmid DNA, which was then transfected into Sf9 cells using Cellfectin II reagent (Invitrogen). PSaV VLPs were expressed in baculovirus-transformed Sf9 insect cells at 27°C and harvested at 5 to 7 days postinfection. A total of 500 ml of VLP-containing supernatants was concentrated by CsCl gradient ultracentrifugation as described below, and the purified VLPs were then stored in aliquots at −80°C or immediately used for the pulldown assay described below.

### Purification of PSaVs and VLPs by CsCl gradient centrifugation.

PSaV particles and VLPs were purified by CsCl gradient ultracentrifugation as described previously (57). Briefly, infected cells harvested at 72 h p.i. for PSaV and at 5 to 7 days p.i. for PSaV VLPs were freeze-thawed three times, and cell debris was removed by centrifugation at 2,890 × *g* for 10 min at 4°C. A total of 500 ml of virus-containing supernatants was concentrated by centrifugation at 141,000 × *g* for 20 h at 4°C using an 80S rotor (Hitachi Koki Himac CP100WX ultracentrifuge). After discarding the supernatants, the virus- or VLP-containing pellets were resuspended in TNE buffer (50 mM Tris-HCl, 100 mM NaCl, 100 mM EDTA, pH 7.5), layered over a 29 to 41% preformed discontinuous CsCl gradient, and then centrifuged at 284,000 × *g* for 20 h at 4°C using a 40S rotor (Hitachi). After collecting the banded PSaV particles or VLPs by puncturing the side of the tube with a needle, each suspension was diluted in distilled water and further purified by ultracentrifugation at 284,000 × *g* for 20 h at 4°C with a 40S rotor (Hitachi). The purified viruses were dialyzed into 0.1 M sodium bicarbonate buffer (pH 8.3) and then stored in aliquots at −80°C until they were used.

### Labeling of PSaV particles with AF594 and calculation of virus particle numbers by TEM.

The procedure for labeling PSaV particles was based on a protocol described previously (57). Briefly, CsCl density gradient-purified PSaV particles (10 mg at 1 mg ml^−1^) in 0.1 M sodium bicarbonate buffer (pH 8.3) were labeled with a 0.1-fold molar concentration of AF594 succinimidyl ester (1 mg at 1 mg ml^−1^ in dimethyl sulfoxide [DMSO]), mixed thoroughly by vortexing for 30 s, and incubated for 1 h at room temperature with continuous stirring. The AF594-labeled PSaV particles were purified again by CsCl density gradient centrifugation, dialyzed against virion buffer, and stored in 2-μg aliquots at −80°C as described above (68). After confirming the specific labeling of viral protein by Western blot analysis and Coomassie blue staining, the number of AF594-labeled PSaV particles was calculated as described previously (69, 70). Briefly, CsCl-purified AF594-labeled PSaV particles were mixed with an equal volume of a suspension of 120-nm latex beads (Sigma-Aldrich) and then applied to the grids. The virus particles were stained with 3% phosphotungstic acid at pH 7 at 20°C for 3 min and observed under an H-7650 transmission electron microscope (TEM) (Hitachi, Tokyo, Japan). The numbers of virus particles and beads were calculated from at least 10 randomly chosen squares on the grid. The total virus count was calculated as the ratio of the virus particle number to the number of latex particles multiplied by the known latex particle concentration per milliliter. The infectivity of the purified Alexa Fluor-labeled PSaV was examined by plaque and qPCR assays as described below.

### Measurement of TER.

The degree of tightness or leakiness of TJs was evaluated by measuring TER across LLC-PK monolayers grown on transwell inserts (Corning, Corning, NY, USA) as described previously (5, 71). Briefly, confluent LLC-PK cells were first inoculated with PSaV strain Cowden (MOI = 50) or PSaV VLPs (4 μg/ml) with or without GCDCA or treated with GCDCA alone before measuring TER using a voltmeter (Millicell ERS-2). In the mock-treated and/or mock-inoculated group, only medium was added to each well. In each sample, the medium was changed before obtaining the measurement. The probe was inserted into the transwell filters, and values were recorded once TER had stabilized. TER values were calculated by subtracting the resistance of the blank from the sample well resistance and multiplying the membrane diameter of the transwell filter culture plate by the resistance in each sample (5, 72).

### Paracellular-flux assay.

Paracellular flux was determined as described previously (5, 73). Briefly, confluent LLC-PK cells grown on transwell inserts were treated with MEM only and treated or not with 200 μM GCDCA for 1 h or infected or not with PSaV strain Cowden (MOI = 50) or PSaV VLPs (4 μg/ml) in the presence or absence of 200 μM GCDCA for 1 h. As a positive control, 1.8 mM calcium ion chelating agent, EGTA, was used to treat another set of monolayers for 10 min. Subsequently, 250 μl of the tracer solution (10 μg/ml 4-kDa or 70-kDa FITC-dextran; Sigma-Aldrich) was applied to the apical sides of the confluent LLC-PK cells. In the mock-treated and/or mock-inoculated group, only medium was added to each well. The samples were incubated for 1 h at 37°C, and media from the upper and lower chambers were collected and measured using a fluorometer (FluroMax 2; Horiba, Kyoto, Japan) at 492 nm (excitation) and 520 nm (emission).

### Membrane lipid diffusion assay.

The membrane lipid diffusion assay was carried out as described previously (5, 73). Briefly, confluent LLC-PK cells grown on transwell inserts were mock treated or treated with 1.8 mM EGTA and incubated for 10 min or infected with PSaV strain Cowden (MOI = 50) or PSaV VLPs (4 μg/ml) with or without the presence of 200 μM GCDCA and incubated for 1 h. Bodify FL-C12-sphingomyelin–BSA complex solution (5 nmol/ml; Molecular Probes) was then applied to the apical sides of confluent LLC-PK cell monolayers and incubated on ice for 60 min. In the mock-treated and/or mock-inoculated group, only medium was added to each well. After incubation, the cells were washed twice with cold P buffer (145 mM NaCl, 10 mM HEPES, pH 7.4, 1.0 mM Na pyruvate, 10 mM glucose, and 3.0 mM CaCl_2_) and incubated on ice for 1 h. The membranes from the insert frame were removed, mounted in P buffer on a glass slide, and covered with a rectangular coverslip. Samples were analyzed immediately by z-sectioning with an LSM 510 confocal microscope using LSM software (Carl Zeiss, Jena, Germany).

### Immunofluorescence assay.

LLC-PK monolayers grown on 8-well chamber glass slides were infected or not with PSaV strain Cowden (MOI = 50) and incubated on ice for 1 h. Entry was initiated by incubating the cells at 37°C in a 5% CO_2_ atmosphere for 30 or 60 min. To determine the distribution of the basolateral membrane marker Na^+^K^+^-ATPase, confluent LLC-PK cells grown on transwell inserts were mock treated or treated with 1.8 mM EGTA and incubated for 10 min or infected with PSaV strain Cowden (MOI = 50) or PSaV VLPs (4 μg/ml) with or without the presence of 200 μM GCDCA and incubated for 1 h. The cells were fixed for 10 min with 4% paraformaldehyde on ice and permeabilized for 2 min with 0.01% Triton X-100 on ice. Afterward, the cells were washed twice with phosphate-buffered saline (PBS) containing 1% FBS and incubated overnight with antibodies against each TJ protein. The secondary antibodies, goat anti-rabbit or anti-mouse conjugated to AF488 or AF647 (diluted 1:100), were incubated for 1 h at 20°C in the dark. Samples were mounted with SlowFade Gold antifade reagent containing DAPI for nuclear staining. The cells were observed by x-sectioning or z-sectioning under an LSM 510 confocal microscope and analyzed with LSM software (Carl Zeiss). The internalization of TJ proteins was quantified by counting the cells with occludin in the cytoplasm and expressed as a percentage of the total number of cells.

### Virus internalization assay using AF594-labeled PSaV particles.

LLC-PK cells transfected or not with siRNA or plasmids were cultured in 8-well chamber slides with or without AF594-labeled PSaV particles (400 particles per cell) for 30 min at 4°C. The cells were then shifted to 37°C for the indicated times to allow virus entry to proceed. To examine the colocalization of AF594-labeled PSaV particles with each TJ protein (occludin, claudin-1, JAM-1, or ZO-1) and each endosomal marker (EEA1 or LAMP2), the cells were permeabilized by the addition of 0.01% Triton X-100 in PBS for 2 min on ice, fixed with 4% paraformaldehyde in PBS for 10 min at 4°C, and washed with PBS containing 1% FBS. The cells were then incubated with antibodies against occludin, claudin-1, JAM-1, ZO-1, EEA1, or LAMP2 (1:100 dilution) at 4°C overnight; washed twice with PBS-FBS; and incubated with FITC-conjugated secondary antibodies (1:100 dilution) at 20°C for 1 h. The cells were mounted with SlowFade Gold antifade reagent containing 1× DAPI solution for staining of nuclei. The slides were analyzed immediately by x- and z-sectioning under an LSM 510 confocal microscope using LSM software (Carl Zeiss). The internalization of occludin, claudin-1, JAM-1, or ZO-1 was quantified by counting cells containing each TJ protein in the cytoplasm and expressed as a percentage of the total number of cells.

### Infectivity assay.

Confluent LLC-PK cells in 6-well plates, transfected or not with siRNA, were washed twice with MEM, inoculated with PSaV strain Cowden (MOI = 50), and then adsorbed for 1 h at 37°C as described previously (4, 5, 10). To determine whether antibodies against occludin or claudin-1 reduced virus infectivity by blocking the binding of virus to the cell surface or at a postbinding step, antibodies were applied to confluent LLC-PK cells in 6-well plates before inoculation of the Cowden strain (MOI = 50) or after adsorption of virus for 1 h, as described previously (4, 5, 10). The cells were washed twice with MEM, followed by the addition of 200 μM GCDCA in medium with 2.5% FBS. They were then assayed by qPCR and TCID_50_ assays at various time points as described below.

### Immunoprecipitation and pulldown assays.

To examine direct interactions between PSaV and TJ proteins, we carried out an immunoprecipitation assay as described previously (74). Briefly, LLC-PK cells grown on 6-well plates were infected with PSaV strain Cowden (MOI = 50) for 1 h on ice, washed twice with PBS, and then shifted to 37°C for 30 min as described previously (4, 5, 10). The cells were lysed with nondenaturing lysis buffer (50 mM Tris-HCl, pH 7.4, 150 mM NaCl, 1 mM EDTA), and the lysates were precleared by incubation for 30 min with protein A or G agarose beads at 4°C under gentle rotation. Subsequently, the precleared cell lysates were incubated overnight with antibodies against occludin, claudin-1, JAM-1, ZO-1, or PSaV capsid protein at 4°C. The immune complexes were captured for 4 h by incubation with protein A or G agarose beads at 4°C, and the immunoprecipitated proteins were then evaluated by Western blotting as described below. To confirm the presence of each target protein and PSaV capsid protein in the cell lysates, the lysates of PSaV-infected LLC-PK cells were directly analyzed by Western blotting using the relevant antibody.

A pulldown assay with CsCl-purified PSaV particles or CsCl-purified PSaV VLPs and GST-tagged occludin using MagneGST glutathione particles (Promega) was performed following the manufacturer’s instructions. Briefly, purified PSaV (400 particles per cell) or purified PSaV VLPs (20 μg/ml) were incubated overnight with GST-tagged occludin (1 μg/ml) at 4°C. Afterward, each mixture was incubated for 4 h with prewashed MagneGST beads at 4°C. MagneGST beads alone and MagneGST beads incubated with GST-tagged occludin (1 μg/ml) served as negative and positive controls, respectively. The MagneGST beads were pulled down using magnetic bars, and then the supernatants were discarded. The pelleted beads were washed twice with washing buffer, and the pulled-down proteins were evaluated by Western blotting as described below.

### Blocking assay.

The antibody blocking assay was performed as described previously (10). To determine if the antibody treatment affected virus binding, confluent LLC-PK cells were grown in 24-well plates and then incubated for 1 h at 4°C with 1 μg/ml of occludin or claudin-1 MAbs or 20 μg/ml of a control IgG antibody. The cells were washed twice with MEM to discard the unbound antibody and incubated for 1 h with PSaV strain Cowden (4 × 10^2^ particles/cells) at 4°C. Afterward, the cells were washed twice with MEM to remove the unbound PSaV and then cultured as described above for various times. Another set of LLC-PK cells were inoculated with PSaV for 1 h at 4°C and then washed twice with MEM to remove unbound virus. This was followed by incubation with MAbs against occludin or claudin-1 or 20 μg/ml of a control IgG antibody. The cells were washed twice with MEM to remove the unbound virus and then incubated for various times. Samples were harvested and processed for qRT-PCR and TCID_50_ assays as described below.

### Construction of mutant plasmids.

Wild-type pmRFP-occludin plasmid (a generous gift from Jerrold R. Tuner, University of Chicago) was used to construct the plasmid encoding the wild-type occludin tag with GFP at the N terminus. Using a primer pair specific for the coding region of occludin (Table 1), the occludin gene was amplified by PCR from a plasmid encoding wild-type pmRFP-occludin. After enzymatic digestion and purification, the occludin gene was inserted into a pEGFP-C1 expression plasmid (here designated GFP-WT-OCC). Using the plasmid encoding GFP-WT-OCC, two mutant plasmids (GFP-M1-OCC and GFP-M2-OCC) containing four silent-mutation sites corresponding to each siRNA were generated by site-directed mutagenesis (GeneTailor site-directed mutagenesis; Invitrogen) using primer-targeting mutation sites according to the manufacturer’s instructions. The mutation sites are shown in Fig. 7A.

### Transfections of siRNAs and plasmids.

Transfection of siRNAs or plasmids was performed in 70 to 80% confluent LLC-PK cells plated in 12-well plates or 8-well chamber slides using Lipofectamine 3000 reagent (Invitrogen) according to the manufacturer’s instructions. LLC-PK cells transfected with the above-mentioned plasmids were used for entry assays at 24 h hpt, whereas siRNA-transfected cells were used at 24 hpt or 48 hpt, depending on the silencing of target protein expression. Cells were washed twice with PBS for various times for the virus infectivity assay or processed immediately for the virus internalization assay described below.

### Infection of occludin-transfected CHO cells.

CHO cells grown in 6-well plates or 8-well chamber slides were transduced with 1 μg pmRFP1-occludin or pCDNA3.1 plasmid. After 24 h, the cells were inoculated with purified PSaV at an MOI of 50. After 2 h of virus adsorption, the cells were washed twice with PBS, and maintenance medium, which contained 200 μM GCDCA and 2.5% FBS, was added, and the process was allowed to proceed. Samples were harvested at 24, 48, and 72 h p.i. The samples were processed for CM, Western blotting, and qPCR analyses as described below.

### RNA isolation and qPCR analysis.

RNA extraction and qPCR analysis were performed as described previously (75). Briefly, LLC-PK cells transfected or not with siRNA or treated or not with antibody and infected or not with AF594-labeled or nonlabeled PSaV strain Cowden (MOI = 50) were incubated for various times and freeze-thawed three times, and cell debris was removed by centrifugation at 2,890 × *g* for 10 min at 4°C. Total RNA was extracted from 200 μl of virus-containing supernatant using a PureLink RNA Mini Kit (Ambion Life Technologies) following the manufacturer’s instructions. The RNA concentrations were read at 260 nm using a BioPhotometer Plus (Eppendorf, Hamburg, Germany), and 1 μg of RNA was subsequently transcribed using random hexamers (Promega). The 25-μl-volume qPCR mixture consisted of 10 pmol forward and reverse primers, cDNA, and Topreal qPCR 2× premix (Enzynomics, Daejon, South Korea). The amplification conditions used for the VPg primer set comprised denaturation at 95°C for 10 min, followed by 40 cycles of denaturation at 95°C for 10 s, primer annealing at 60°C for 20 s, and extension at 72°C for 20 s. Tenfold dilutions of a known amount of pCV4A was used to generate the standard curve to calculate the copy number of VPg in the samples. Each sample was assayed in triplicate on the same qPCR plate in two independent experiments. Non-template and non-reverse transcriptase reactions were analyzed routinely as negative controls. Data were collected using Rotor Gene 6000 software (Corbett Research, Mortlake, Australia).

### TCID_50_ assay.

The TCID_50_ assay was performed as described previously (51). Briefly, 10-fold serial dilutions of virus-containing supernatant obtained from cells by three freeze-thaws or from CsCl density gradient-purified and AF594-labeled PSaV particles were prepared in MEM supplemented with 200 μM GCDCA. From these dilutions, 200 μl was inoculated into monolayers of LLC-PK cells grown on 96-well plates and incubated at 37°C in a 5% CO_2_ atmosphere. Virus titers were determined using the Reed-Muench method (77) 6 days postinfection and expressed as TCID_50_ per milliliter (76).

### Plaque assay.

The plaque assay was performed as described previously (51). Briefly, monolayers of LLC-PK cells grown on 6-well plates were inoculated with 800 μl of diluted virus stock, AF495-labeled PSaV particles, or medium alone (37°C; 3 h) and gently shaken for 1 h with occasional shaking to allow virus adsorption. Afterward, the inoculated cell monolayers were washed three times with PBS and overlaid with 1.3 % (wt/vol) Avicel-containing MEM supplemented with 2.5 % (vol/vol) FBS, 200 μM GCDCA, 0.225 % (vol/vol) sodium bicarbonate, and penicillin-streptomycin. The plates were incubated at 37°C for 4 days, and the Avicel mixture was removed. The cells were fixed with 10 % (vol/vol) formaldehyde solution in 1× PBS for 30 min and stained with 1.6 % (wt/vol) methylene blue. The plates were washed with distilled water until the desired staining intensity was achieved. Plaques were counted, and PFU were calculated by the following formula: number of PFU = number of plaques/dilution factor × volume of virus added to the well.

### Western blot analysis.

The cell lysates, immunoprecipitated proteins, and pulled-down proteins prepared as described above were denatured and resolved in an SDS-PAGE gel. The resolved proteins were transferred to nitrocellulose blotting membranes (GE Healthcare Life Sciences). The membranes were blocked for 1 h at room temperature with Tris-buffered saline containing 5% skim milk before they were incubated overnight at 4°C with the indicated primary antibodies. The bound antibodies were developed by incubation with HRP-labeled secondary antibody, and immunoreactive bands were detected by enhanced chemiluminescence (ECL) (Dogen, Seoul, South Korea) using the Davinch-K imaging system (Youngwha Scientific Co., Ltd., Seoul, South Korea). To confirm equal protein loading, the blotting membranes were reprobed with an antibody against GAPDH, and the reactivity was compared with the intensities of the target bands.

### Statistical analyses and software.

Statistical analyses were performed on the results of triplicate experiments using GraphPad Prism software version 5.03 (GraphPad Software Inc., La Jolla, CA, USA) and a one-way analysis of variance (ANOVA) test. *P* values of less than 0.05 were considered statistically significant. The figures were generated using Adobe Photoshop CS3 and Prism 5 version 5.03.
